# Formation of ferromagnetic molecular thin films from blends by annealing

**DOI:** 10.3762/bjnano.8.146

**Published:** 2017-07-14

**Authors:** Peter Robaschik, Ye Ma, Salahud Din, Sandrine Heutz

**Affiliations:** 1 Department of Materials and London Centre for Nanotechnology, Imperial College London, Exhibition Rd, London SW7 2AZ, United Kingdom; 2 Kurt J. Lesker Company, Sidney Little Rd, St Leonard’s on Sea TN38 9PU, United Kingdom

**Keywords:** co-deposition, molecular spintronics, organic thin films, phthalocyanines, tetracyanoquinodimethane (TCNQ)

## Abstract

We report on a new approach for the fabrication of ferromagnetic molecular thin films. Co-evaporated films of manganese phthalocyanine (MnPc) and tetracyanoquinodimethane (TCNQ) have been produced by organic molecular beam deposition (OMBD) on rigid (glass, silicon) and flexible (Kapton) substrates kept at room temperature. The MnPc:TCNQ films are found to be entirely amorphous due to the size mismatch of the molecules. However, by annealing while covering the samples highly crystalline MnPc films in the β-polymorph can be obtained at 60 °C lower than when starting with pure MnPc films. The resulting films exhibit substantial coercivity (13 mT) at 2 K and a Curie temperature of 11.5 K.

## Introduction

Controlling the structure of molecular thin films is of great interest for their application in optoelectronic and spintronic devices [1–2]. One of the key physical properties determining the performance is crystallinity, which significantly influences electron/hole mobility and magnetic coupling [3–4]. We recently reported on an approach to achieve crystalline porphyrazine films, which are amorphous on non-interacting substrates, by depositing the molecules on a 3,4,9,10-perylenetetracarboxylic dianhydride (PTCDA) surface, leading to a transformation from paramagnetic to antiferromagnetic behaviour [5]. Another promising class of molecules are phthalocyanines (Pc) that can be easily incorporated in thin films that exhibit outstanding semiconducting and magnetic properties [6].

Phthalocyanines feature different polymorphs, often defined by the angle formed by the molecule and the stacking axis, φ, which can be controlled via different temperature treatments. For instance, at room temperature, films commonly adopt the α-phase (φ = 65°) and can be transformed to the thermodynamically stable β-phase (φ = 45°) by annealing in vacuum at 330 °C [7–9]. However, such temperatures are too high for most flexible substrates, and therefore limit one of the main fabrication advantages of molecular materials.

The β-phase is particularly attractive for MnPc, where it leads to ferromagnetism in polycrystalline powders and single crystals [10–11]. Due to the arrangement in molecular columns with a stacking angle of 45° the Mn ion lies directly underneath a nitrogen atom of the nearest-neighbouring MnPc molecule. The magnetic interaction has been attributed to superexchange [11–13], although more recent results highlight that indirect exchange also contributes to the mechanism [14].

Here we develop a strategy to lower the phase-transition temperature of MnPc by 60 °C by blending the MnPc film with TCNQ in the starting films deposited at room temperature. Optical microscopy and X-ray diffraction (XRD) are used to identify the phase transition by investigation of the surface morphology and the structure of the films, while FTIR spectroscopy provides information about the chemical composition and structure of the films. Furthermore superconducting quantum interference device (SQUID) magnetometry measurements reveal the ferromagnetic behaviour of the β-MnPc films, which exhibit remarkable coercivity. The opening of the hysteresis loop is preserved at temperatures up to 10 K, and the Curie temperature as determined by susceptibility measurements is 11.5 K. The combination of new processing methodologies with attractive magnetic properties will have important implications for spintronics.

## Results and Discussion

### Sample preparation

**Figure 1:**
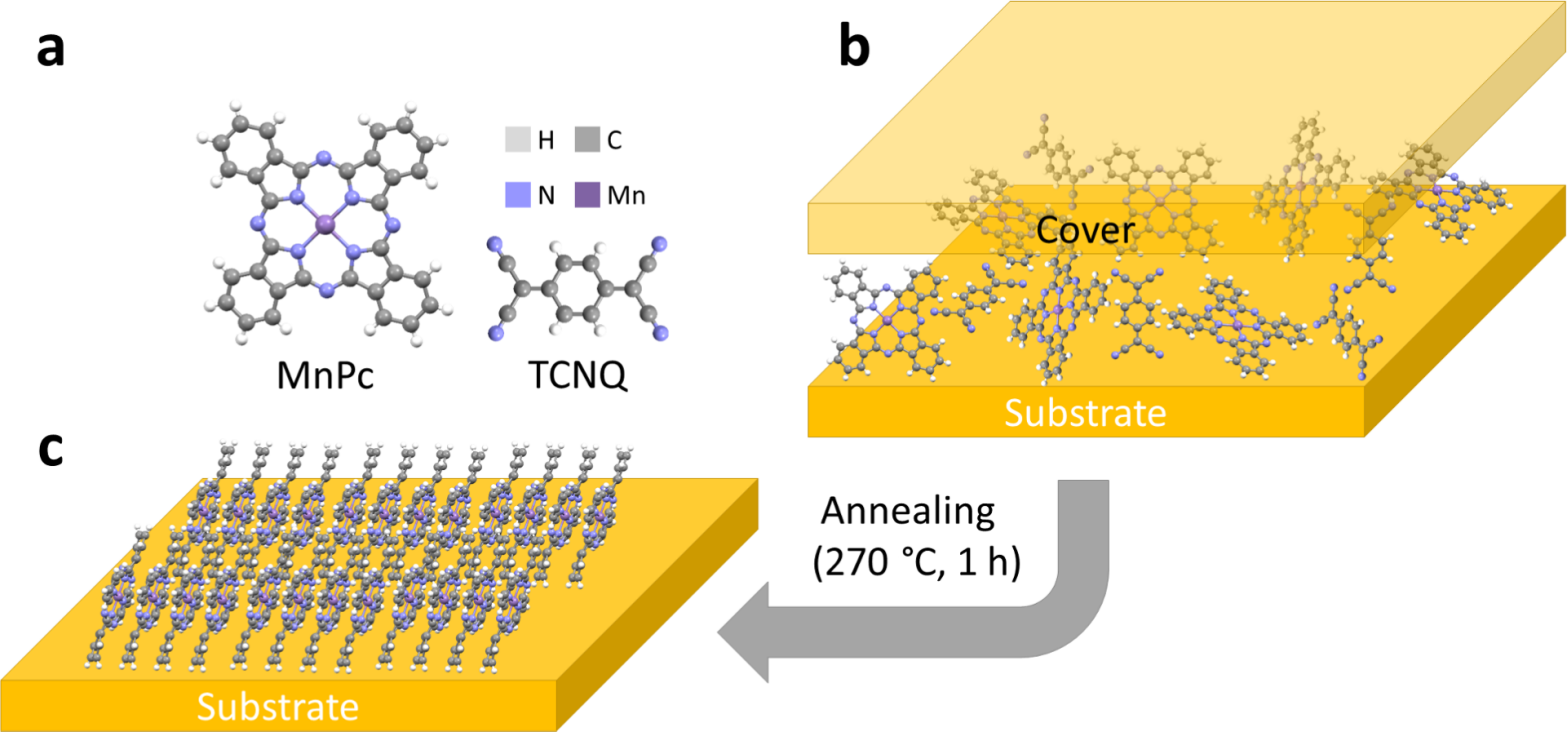
(a) Manganese phthalocyanine (MnPc) and tetracyanoquinodimethane (TCNQ) molecules. (b) Annealing procedure applied to the blended thin films prepared by OMBD. (c) Well-ordered β-MnPc film after annealing.

Blended films comprising MnPc and TCNQ (Figure 1a) have been prepared by organic molecular beam deposition (OMBD). A one-to-one ratio of the desired molecules in the films was obtained with a deposition rate of 0.5 Å/s and a thickness of 100 nm for MnPc. Due to its higher molecular density TCNQ is deposited at a lower rate of 0.22 Å/s leading to a thickness of 44 nm. Hence the total thickness of the blended film amounts to 144 nm.

Figure 1b shows the annealing procedure in which the blended film is covered (see Experimental section) without applying any additional force and put inside a tube furnace. Once the tube is pumped down and flushed with nitrogen at a flow rate of 150 sccm for at least one hour the furnace is set to 270 °C and left dwelling for one hour after the final temperature is reached. During this process the TCNQ molecules can slowly escape the film due to their low sublimation temperature of approximately 100 °C [15].

We compare these films with neat MnPc films, prepared using sublimation of MnPc on substrates at room temperature at a rate of 0.5 Å/s up to a thickness of 100 nm, and subjected to subsequent annealing procedures as for the blends. As will be discussed later we believe that the emerging vacancies in the mixed films generate sufficient free volume around the MnPc molecules for a rearrangement to the thermodynamically stable β-phase (Figure 1c), which normally forms above 300 °C [7–8].

### Film morphology, structure and composition

**Figure 2:**
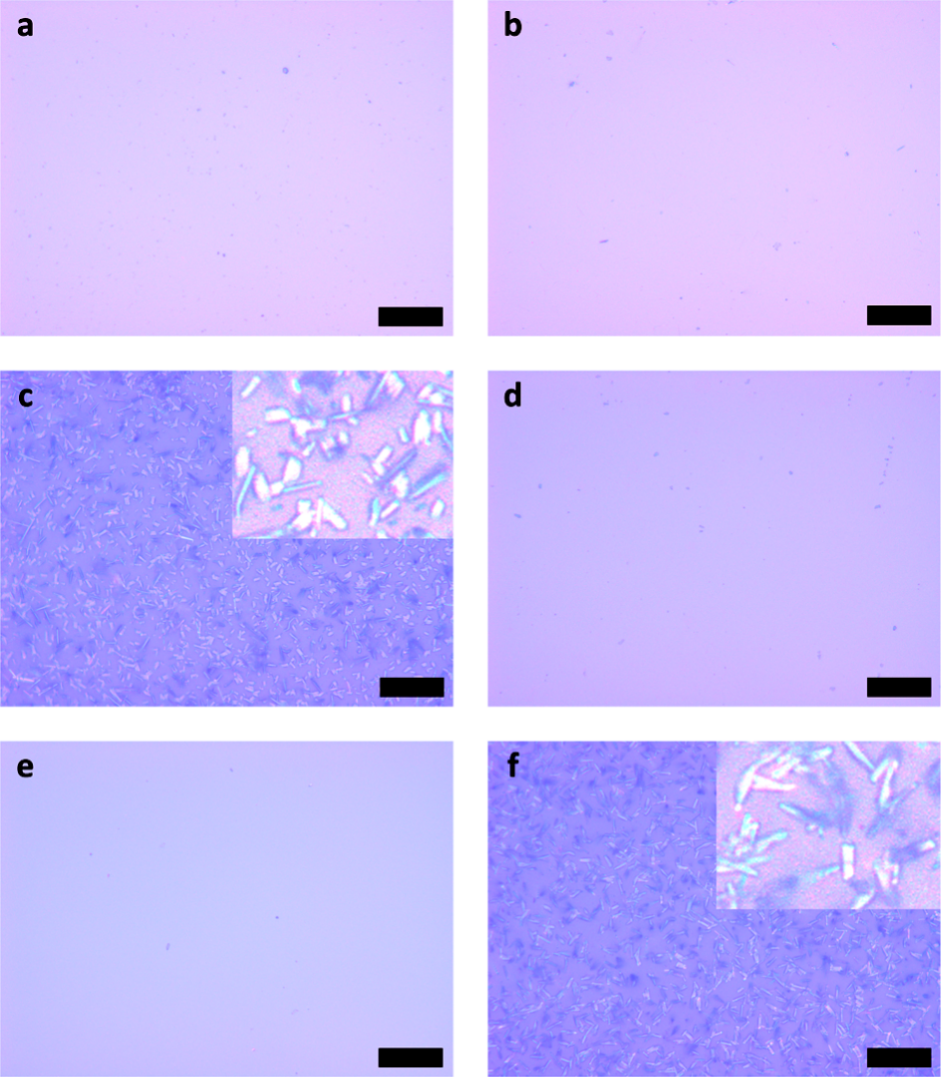
Optical micrographs for molecular thin films grown on glass substrates. (a) α-MnPc film grown at room temperature. (b) MnPc film annealed at 270 °C without cover. (c) β-MnPc film formed by annealing of the α-MnPc sample at 330 °C with cover. (d) MnPc:TCNQ film grown at room temperature. (e) MnPc:TCNQ film annealed at 270 °C without cover. (f) β-MnPc film formed by annealing of a MnPc:TCNQ sample at 270 °C with cover. The scale bars correspond to a length of 20 µm for the images and 5 µm for the insets.

Optical micrographs in Figure 2 reveal the surface morphology of the organic films following different thermal treatments. For comparison the neat MnPc film deposited on glass kept at room temperature (Figure 2a) is shown with a very smooth surface and a grain size that is below the detection limit of the optical microscope. Annealing at 270 °C without a cover for one hour (Figure 2b) does not affect the surface morphology. However, increasing the temperature to 330 °C and covering the films (Figure 2c) leads to the formation of larger crystallites.

Similar results are obtained for the mixed films (Figure 2d–f), although annealing at a temperature of 270 °C already allows the molecules to rearrange into large elongated crystallites. This was observed previously for iron phthalocyanine (FePc) thin films deposited at different substrate temperatures [16]. However, in that case the length of the major axis was found to be 200 nm at a temperature of 260 °C. The crystallites in our MnPc films reach a size of up to 10 µm for their longer axis, which is attractive for high coercivity in magnetism and improved charge transport along the crystallites.

**Figure 3:**
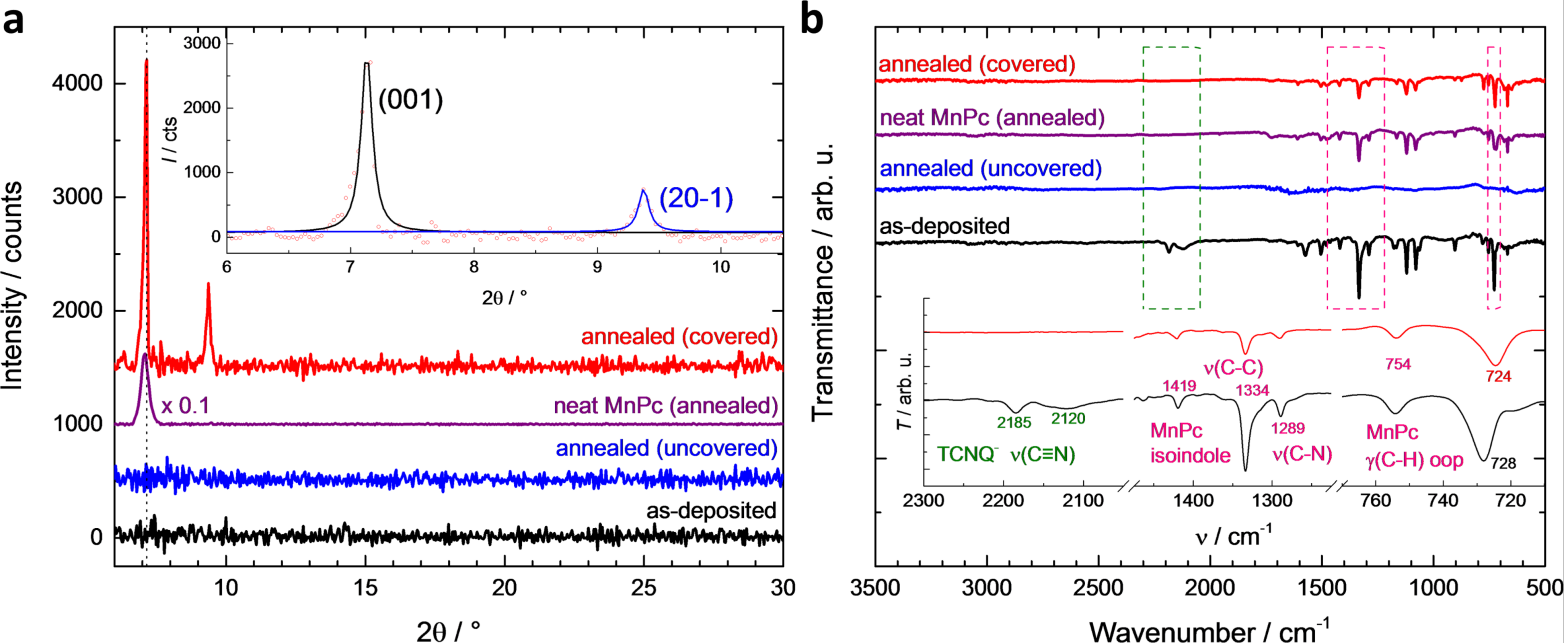
(a) X-ray diffraction patterns. For an as-deposited 144 nm thick MnPc:TCNQ film on silicon (black) no diffraction peaks are observed. Annealing without cover (blue) leads to similar results, whereas the covered film (red) exhibits two diffraction peaks with high intensity. For comparison the XRD pattern of a neat MnPc film (purple) that was annealed at the same temperature is shown. All films were annealed at a temperature of 270 °C. (b) FTIR spectra for the same films deposited on KBr substrates. The green frame highlights the range of 2050–2300 cm^−1^ where the ν(C≡N) stretching peaks for TCNQ appear. The pink frames show the area of the MnPc isoindole vibrations around 1225–1475 cm^−1^ and the γ(C-H) out-of-plane deformation of the MnPc ligand at 710–770 cm^−1^, respectively. The inset shows specific regions in the mixed films (colour coding as in main graph).

Results from optical microscopy can be refined by X-ray diffraction (XRD). Figure 3a shows the XRD patterns of the as-deposited MnPc:TCNQ film on silicon as well as the films that have been annealed with and without cover. The sample without any heat treatment appears entirely amorphous showing no peaks in a range up to 2θ = 30°, where phthalocyanine fingerprints usually appear [4]. Annealing at a temperature of 270 °C without a cover also leads to a featureless XRD pattern. However, as will be shown by FTIR this is due to the absence of any thin film rather than to its amorphous nature.

The annealed MnPc:TCNQ films that were additionally covered, however, show two high intensity peaks at 2θ = 7.13° and 9.37°, which correspond to the diffraction from the (001) and (20−1) planes of β-MnPc [17–18]. We note that the (20−1) plane is not usually observed in β-MnPc films obtained by annealing of the α-phase and its observation indicates a reduced texture. This is presumably due to the disordered nature of the starting film in this case, in contrast to the case of highly oriented α-phase film. The inset of Figure 3a reveals the sharp nature of both peaks and fits using the Lorentz function result in full width at half maximums (FWHM) of 0.11° and 0.10°, respectively. From the obtained FWHMs we can estimate the grain dimensions, τ, out of the sample plane by using the Scherrer equation [19]:

[1]
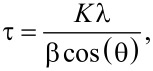


where *K* is the dimensionless shape factor, *λ* is the wavelength of the X-ray source, β is the FWHM and θ is the Bragg angle. Calculations using *K* = 1 give a grain size of 84 ± 9 nm for the β-MnPc film, which is in line with the equivalent film thickness of MnPc in the blended film prior to annealing.

By comparison a neat 100 nm thick MnPc film annealed at 270 °C for one hour shows only one broad diffraction peak at 2θ = 7.09°, which most likely combines the contributions of the (100) and (001) planes of the α- and β-polymorphs, respectively [17,20], assuming that MnPc is isostructural with CuPc. This represents an intermediate case with a partial transformation from α- to β-phase as shown earlier for metal-free phthalocyanine thin films where Yim et al. proposed a phase-transition mechanism that involves tilting of the α-phase molecular columns to form the β-phase [9]. Therefore, for the neat MnPc film the thermal energy provided is not sufficient to transform the entire film from α- into β-phase. Due to the combination of both α- and β-phases, the diffraction peak has been fitted to two Lorentz functions centred at 2θ = 7.03° and 7.16° leading to FWHMs of 0.20° and 0.21°, respectively. The values correspond to a grain size of 45 ± 5 nm, which is in agreement with the values of phthalocyanine films previously deposited at room temperature [16,21], correlates with the smooth morphology and is much lower than the value of the β-MnPc films generated from the blended film.

To further investigate the phase transformation we have conducted FTIR spectroscopy measurements (Figure 3b) on the same films deposited on KBr substrates. The ν(C≡N) stretching peaks of TCNQ for the as-deposited blended film can be found at 2185 and 2120 cm^−1^, respectively. This finding is in contrast to the peak at 2228 cm^−1^ for the neutral TCNQ [22] and suggests charge transfer (CT) from the Mn ion of the phthalocyanines to the TCNQ molecules forming Mn^3+^ and TCNQ^−^. Similar CT was previously observed for MnPc/F_4_TCNQ films by Rückerl and collaborators [23]. Both peaks vanish for every annealed sample proving that all TCNQ molecules sublime during the annealing process. The ν(C–C) (1429 and 1334 cm^−1^) and ν(C–N) (1289 cm^−1^) vibrations of the MnPc isoindole [24] are preserved for the annealed neat film and the annealed mixed film with cover. However, for the MnPc:TCNQ blended film that was annealed without cover it seems that both phthalocyanine and TCNQ molecules sublime. Furthermore, for the covered sample, one peak of the γ(C–H) out-of-plane deformation of the MnPc ligand shows a shift from 728 to 724 cm^−1^ and can be attributed to phase transformation to the β-phase [25].

We believe that the successful crystallisation of the β-phase MnPc at lower temperatures compared to the α→β phase transition thanks to the blending with sacrificial TCNQ molecules is due to two main factors. Firstly, the presence of TCNQ hinders crystallisation of the MnPc molecules, preventing them from forming strong intermolecular bonds and enabling higher mobility for crystallisation into the thermodynamically stable β-phase. This is substantiated by the observation in FTIR of the sublimation of MnPc molecules, which are not stabilised by the crystal lattice, in the uncovered blend film at 270 °C whereas neat α-phase films remain on the substrate under the same conditions. Secondly, when the blend film is covered, MnPc molecules cannot sublime as readily, but TCNQ molecules can diffuse out of the film due to their small size and lower sublimation temperature, creating vacant space which allows the MnPc molecular columns to arrange into the β-phase.

So the co-deposition of phthalocyanines with small molecules could allow for the formation of highly oriented molecular films at lower process temperatures. This is crucial for later integration into electronic and spintronic devices, especially on flexible substrates.

### Magnetic characterisation

**Figure 4:**
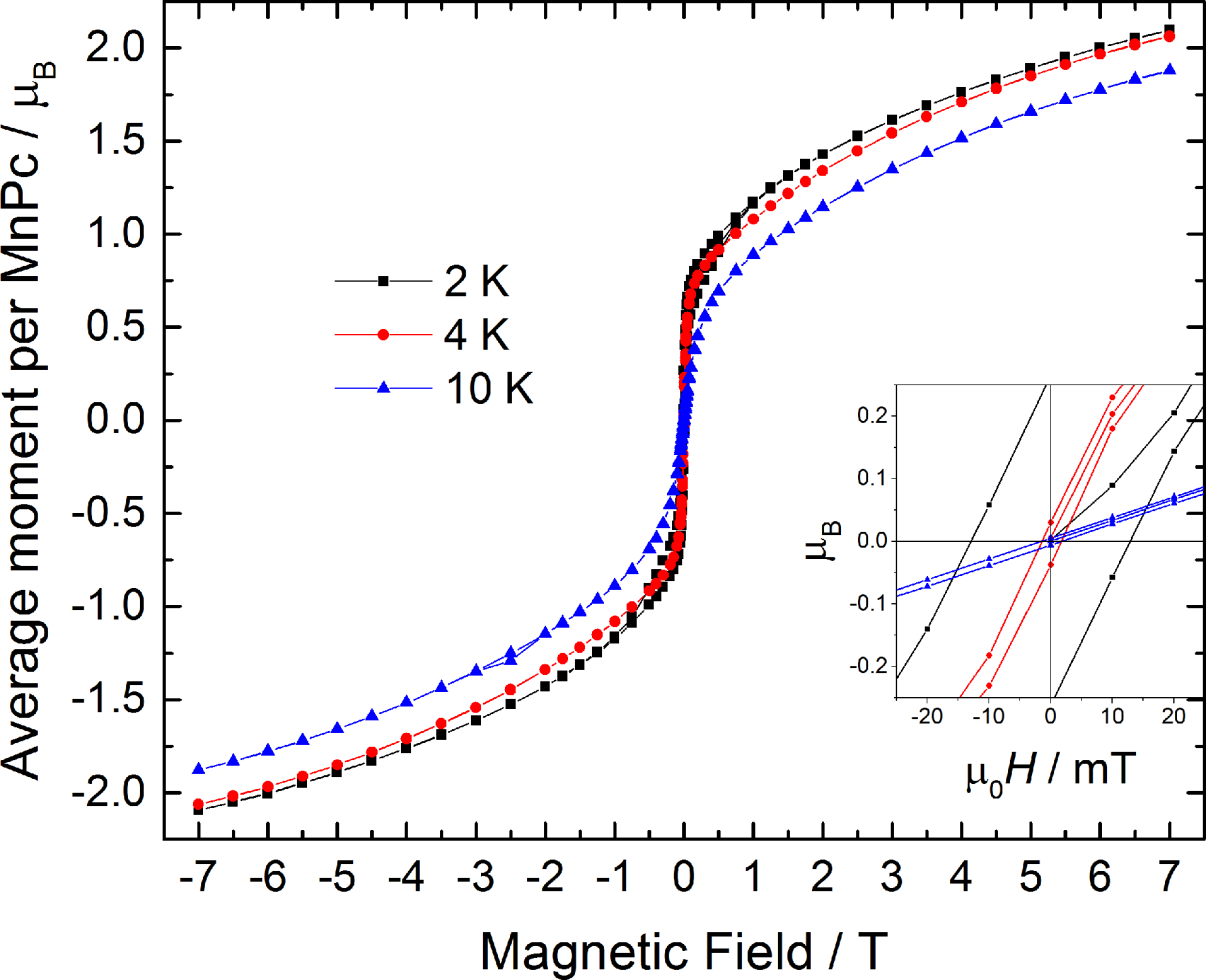
Magnetic hysteresis loops of a MnPc thin film on Kapton obtained from annealing of a MnPc:TCNQ blend. The inset shows that the film exhibits a coercivity of up to 13 mT at 2 K.

As expected from optical microscopy and XRD the β-MnPc film formed by annealing of the MnPc:TCNQ utilising a cover exhibits remarkable magnetic properties. Figure 4 shows magnetic hysteresis loops of a MnPc film on Kapton at different temperatures. A substantial coercivity of 13 mT, which coincides with the 15 mT found for MnPc powder annealed in Ar atmosphere [26], can be detected at 2 K and an opening of the hysteresis is preserved up to a temperature of 10 K. The magnetisation increases rapidly until a field of 100 mT is reached and is not saturated at the maximum field of 7 T. This is in line with earlier reports on β-MnPc powder and single-crystal samples [10–11,27–28]. However, to our knowledge no reports on the coercivity in β-MnPc thin films from OMBD have been made. The magnetic moment at 7 T amounts to 2.1 μ_B_ per MnPc molecules, which is below the expected value of 3 μ_B_ for a system with *S* = 3/2. This can be explained by the absence of magnetic saturation at the maximum available field and the reduction of magnetic moment due to crystal field effects [29]. Due to the observation of a reasonable magnetic moment per MnPc we can further confirm that no significant amount of phthalocyanine molecules have been sublimed during the annealing process.

**Figure 5:**
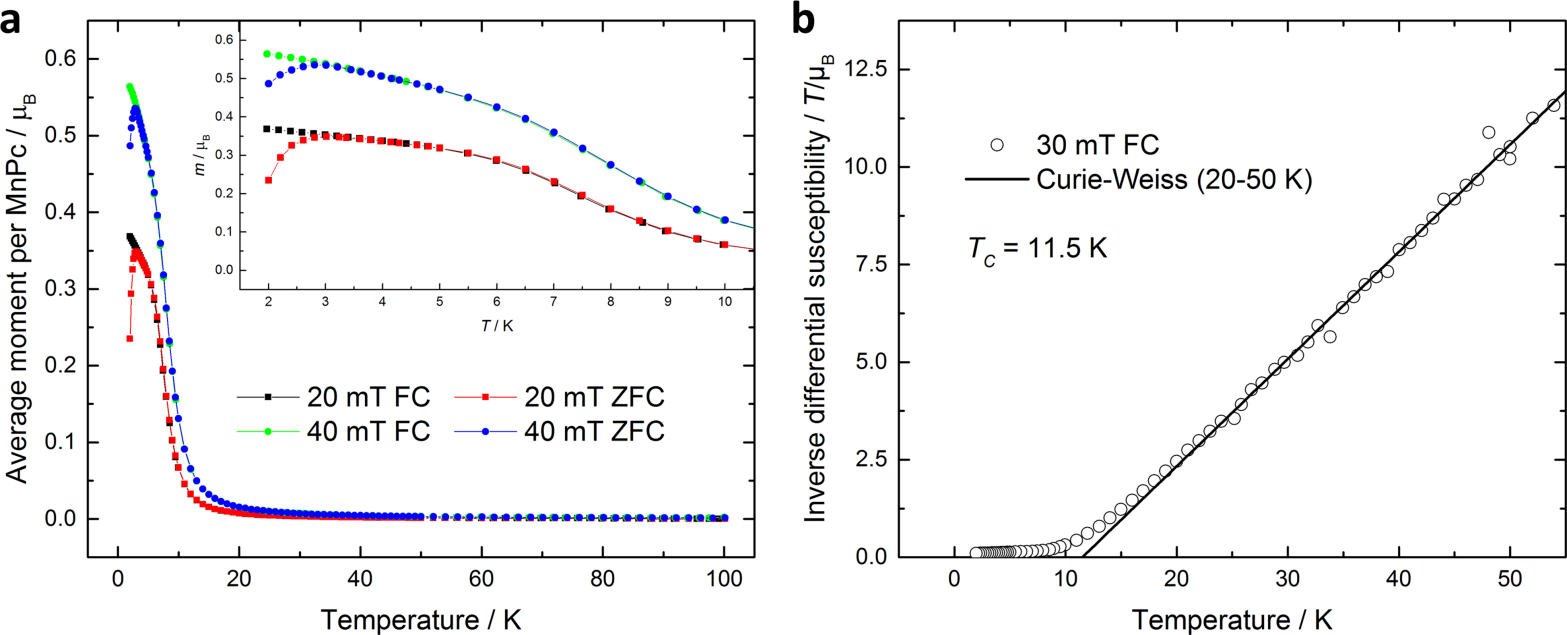
(a) Magnetisation as a function of the temperature at fields of 20 mT and 40 mT using both FC and ZFC protocols. (b) Inverse differential susceptibility calculated from the magnetisation measurements showing a Curie temperature of 11.5 K.

For further analysis the temperature dependence of the magnetisation (Figure 5a) has been measured at fields of 20 and 40 mT for both field cooled (FC) and zero-field cooled (ZFC) protocols. The average magnetic moment per MnPc molecule increases rapidly below 20 K and shows a bifurcation in both ZFC measurements at 3 K, whereas a steady increase in the FC data can be observed. This finding coincides with reports on DC and AC susceptibility measurements of pure β-MnPc powder showing a slow relaxation of magnetisation [30–31]. Further studies by Wang and Seehra were able to rule out spin-glass behaviour and found that the zero-field splitting (ZFS) parameter |*D*|/*k*_B_ = 8.3 K is much larger than the coupling constant *J*/*k*_B_ = 2.6 K [32]. According to Moriya, in this case (|*D*|/*J* > 1) long-range ordering is not possible [33], which leads to the assumption that β-MnPc can be treated as a single-chain magnet.

From the temperature-dependent magnetisation values collected at two different fields the inverse differential susceptibility was determined and is shown in Figure 5b. The data follows a linear behaviour at higher temperatures and can be fitted to the Curie–Weiss law in a temperature range from 20 to 50 K. The Curie temperature is found from the intercept with the *y*-axis at 11.5 K and is slightly higher compared to reported values of 6–10 K for β-MnPc crystals [10–11,28,34].

## Conclusion

We report on an approach to fabricate ferromagnetic β-MnPc films by annealing of MnPc:TCNQ blends, which reduces the process temperature by 60 °C compared to the preparation procedure starting from a neat MnPc film. The resulting films form large elongated crystallites with their long axis measuring up to 10 µm. X-ray diffraction studies show that the out-of-plane dimensions of the grains (84 ± 9 nm) are comparable to the target film thickness of 100 nm, which means that no grain boundaries parallel to the substrate plane are expected. Magnetic measurements reveal remarkable ferromagnetic properties with a substantial coercivity of up to 13 mT at 2 K and a Curie temperature of 11.5 K. These findings are significant for a future implementation of magnetic molecular thin films in spintronic devices, especially on flexible substrates that are commonly sensitive to high process temperatures. We anticipate that the methodology for reducing phase-transition temperatures through the blending strategy can be generalised to a wide range of systems, provided the sacrificial molecules used in the blend reduce intermolecular interactions and have a sublimation point below that of the molecule of interest.

## Experimental

Organic thin films have been grown using a SPECTROS deposition chamber by Kurt J. Lesker Company with a base pressure of 3 × 10^−7^ mbar. During the co-deposition in total three quartz crystal microbalances (QCM) were utilised to achieve a one-to-one ratio of the molecules. Two QCMs monitored the deposition rate of each organic source and the combined rate was confirmed by a third QCM next to the substrates.

The annealing procedure was carried out in a furnace (Carbolite HZS-12/900E) fitted with a 150 cm long quartz tube (3 cm outer diameter). For the covered samples the films on glass and silicon substrates were sandwiched between microscope slides, whereas the Kapton samples were covered by another Kapton sheet. The tube was pumped down to 1 × 10^−1^ mbar followed by nitrogen flushing at a flow rate of 150 sccm for at least one hour. After flushing the nitrogen flow was kept at the same rate leading to a pressure of approximately 25 mbar. All three zones of the furnace were set up to 270 °C and once the final temperature was achieved the furnace was left for dwelling for one hour.

The surface morphology was observed with an Olympus BX51 optical microscope using a 100× objective. The crystal structure was studied by using a Panalytical X’Pert PRO MPD X-Ray diffractometer (Ni filtered Cu K_α_ radiation at 40 kV and 40 mA) operated in the θ–2θ mode. The background was subtracted using the Sonneveld method [35].

The composition of the films was investigated in transmission mode utilising a Nicolet iS10 FTIR spectrometer from Thermo Scientific with an optimised spectral range of 7800–350 cm^−1^ and a resolution of 0.4 cm^−1^.

The magnetic measurements were conducted with a Quantum Design MPMS-7 SQUID (superconducting quantum interference device) magnetometer. The films were deposited with a stripe shadow mask (4 × 90 mm^2^) on flexible Kapton foil and rolled into a tube as reported by Heutz et al. [28]. Therefore the size of the Kapton foil was chosen to be larger than the scanning length of the coils in the SQUID to compensate for any background signals that could occur from the substrate.
